# Irrigating the Ear

**Published:** 1887-08

**Authors:** H. E. Stroud

**Affiliations:** Colorado


### Southern Medical Record.

A MONTHLY JOURNAL OF PRACTICAL MEDICINE.

☞All Communications must be addressed to R. C. WORD, M. D., Managing Editor.

"*Quicquid Præcipies Esto Brevis.*"

VOL. XVII. ATLANTA, GA., AUGUST, 1887. No. 8.

### ORIGINAL AND SELECTED ARTICLES.

### IRRIGATING THE EAR.

BY H. E. STROUD, M. D., OF COLORADO.

Sometime since I had a case of inflammation of the middle ear, in which it was necessary to puncture the membrana tympani to prevent rupture; but, after this procedure, the pain, though mitigated, continued severe, and no application would relieve it except hot water thrown into the ear with a syringe. But I soon found it was not easy to keep the water at an even temperature, and it got very tiresome to myself and patient. A thought struck me, which I put into immediate execution: I went to the tin-shop and had a funnel made with a small tip, and to this I attached a yard of rubber tube, and with this I could throw a constant stream of hot water into the ear, by the hour if necessary, without fatigue. I fixed the funnel in a bracket, and a piece of oilcloth around the patient's neck, directing the water, as it ran out of the ear, into a bucket. I found, with the constant current, I could gradually increase the temperature of the water quite high without discomfort and much hotter than would be tolerated with a syringe.

The first time I used this continuous current he went to sleep, and snored in several languages and numerous keys, so complete was the relief.

As I never saw any suggestion of this in any work, I give it for what it is worth, believing that, when it is advisable to use hot ablutions in the ear, it is safer, pleasanter, less trouble, and more efficacious thon the syringe, and in my hands has aborted, with other treatment, what threatened to be a severe and complicated inflammation.

In making examination of the ear, when it is sore and inflamed, a 4-per cent. solution of cocoa is a perfect God-send to the patient, and enables the operator to be more thorough.

